# BMI and plasma lipid levels with risk of proliferative diabetic retinopathy: a univariable and multivariable Mendelian randomization study

**DOI:** 10.3389/fnut.2023.1099807

**Published:** 2023-09-13

**Authors:** Yiyang Shu, Qi Zhou, Yuting Shao, Hui Lin, Shen Qu, Wenting Han, Xiao Lv, Yanlong Bi

**Affiliations:** ^1^Department of Ophthalmology, Tongji Hospital, School of Medicine, Tongji University, Shanghai, China; ^2^Exam Center, School of Medicine, Tongji University, Shanghai, China; ^3^School of Medicine, Tongji Eye Institute, Tongji University, Shanghai, China

**Keywords:** proliferative diabetic retinopathy (PDR), body mass index (BMI), dyslipidemia, Mendelian randomization, causal effect

## Abstract

**Background:**

The study aimed to determine whether a causal effect exists between body mass index (BMI) or plasma lipid levels and proliferative diabetic retinopathy (PDR) risk in humans.

**Methods:**

We utilized univariable (UVMR) and multivariable two-sample Mendelian randomization (MVMR) analyses to confirm the effects of BMI and plasma lipid levels on the risk of PDR. Genetic variants associated with BMI and three plasma lipids were obtained from GWAS summary datasets generated by many different consortia and were deposited in the MR-Base database. The GWAS summary data for PDR from the FinnGen biobank included 2,12,889 participants of European ancestry (8,681 cases and 2,04,208 controls). Inverse variance weighted (IVW) was applied as the main MR analysis. Sensitivity analysis was used to evaluate the robustness of our findings.

**Results:**

In the UVMR analysis, the causal associations of genetically predicted BMI with PDR presented a positive association (OR = 1.120, 95% CI = 1.076–1.167, *P* < 0.001), and the lower HDL-C level was associated with a higher risk of PDR (OR = 0.898, 95% CI = 0.811–0.995, *P* = 0.040). No evidence of an association between LDL-C or TG levels (*P* > 0.05) and PDR risk was found. In the MVMR analysis controlling for the HDL-C level, there was strong evidence for a direct causal effect of BMI on the risk of PDR (OR = 1.106, 95%CI = 1.049, 1.166, *P* < 0.001, IVW). After adjusting for BMI, there was no evidence for a direct causal effect of the HDL-C level on the risk of PDR (OR = 0.911, 95% CI = 0.823, 1.008, *P* = 0.072). Sensitivity analyses confirmed that the results were reliable and stable.

**Conclusion:**

Robust evidence was demonstrated for an independent, causal effect of BMI in increasing the risk of PDR. Further studies are required to understand the potential biological mechanisms underlying this causal relationship.

## Introduction

Diabetes mellitus (DM) is a serious and increasing global health burden. From the 2019 Global Burden of Disease (GBD) study, we found that the all-age prevalence number of DM increased significantly from 1990 to 2019 (1.60 × 10^8^ vs. 4.60 × 10^8^). The increased prevalence of DM worldwide has led diabetic retinopathy (DR) to be a leading cause of vision loss in many countries (1). Among the global 33.6 million adults aged 50 years and older who were blind in 2020, the number of cases of blindness caused by DR was 0.9 million (95% CI 0.6–1.2) (2). DR is a chronic progressive disease caused by damage to the microvasculature of the retina. Proliferative diabetic retinopathy (PDR) is the later stage of DR and is characterized by neovascularization of the retina, which can lead to irreversible vision loss, and its damage cannot be completely cured.

Hyperglycemia is the strongest modifiable risk factor, and people with optimal glycemic control also develop DR. Thus, exploring the role of other modifiable risk factors has become increasingly important in the development and progression of DR, and obesity is one such important factor (3). Some studies (4–7) have shown that dyslipidemia is a risk factor for the development of DR. The Fenofibrate Intervention and Event Lowering in Diabetes (FIELD) study and the Action to Control Cardiovascular Risk in Diabetes (ACCORD) study showed a beneficial effect of fenofibrate on the progression of DR although the mechanism of this effect does not seem to be related to the level of plasma lipids (8, 9). However, the relationship between BMI, plasma lipid level, and PDR may be subject to confounding by unknown factors. Therefore, it is difficult to clarify causal risk factors. Given the lack of evidence, more studies are still needed to clarify the relationship between them.

In general, the gold standard to determine causality is the randomized control trial (RCT); however, RCTs are time-consuming and require a large amount of manpower and resources; moreover, sometimes it is almost impossible to conduct an RCT due to ethical issues (10). Mendelian randomization (MR) is an alternative approach to make inferences about the causal effect between exposures and outcomes by using genetic variants as instrumental variables (IVs) (11). The single nucleotide polymorphisms (SNPs) used as IVs in the MR analysis must meet the following three basic assumptions: (1) the IVs must be associated with the exposure; (2) the IVs must only affect the outcome via the exposure; and (3) there is no factor that can cause both the IVs and the outcome.

To the best of our knowledge, no MR study has investigated the causal relationship between BMI, plasma lipid level, and PDR. The study comprehensively explored whether genetically predicted BMI and plasma lipid levels are risk factors for PDR outcomes to further examine these associations using the MVMR method to rule out pleiotropy. This study aimed to help clarify the risk factors underlying the PDR and assist in the development of future prevention and intervention strategies.

## Materials and methods

### Study design

**Figure 1:**
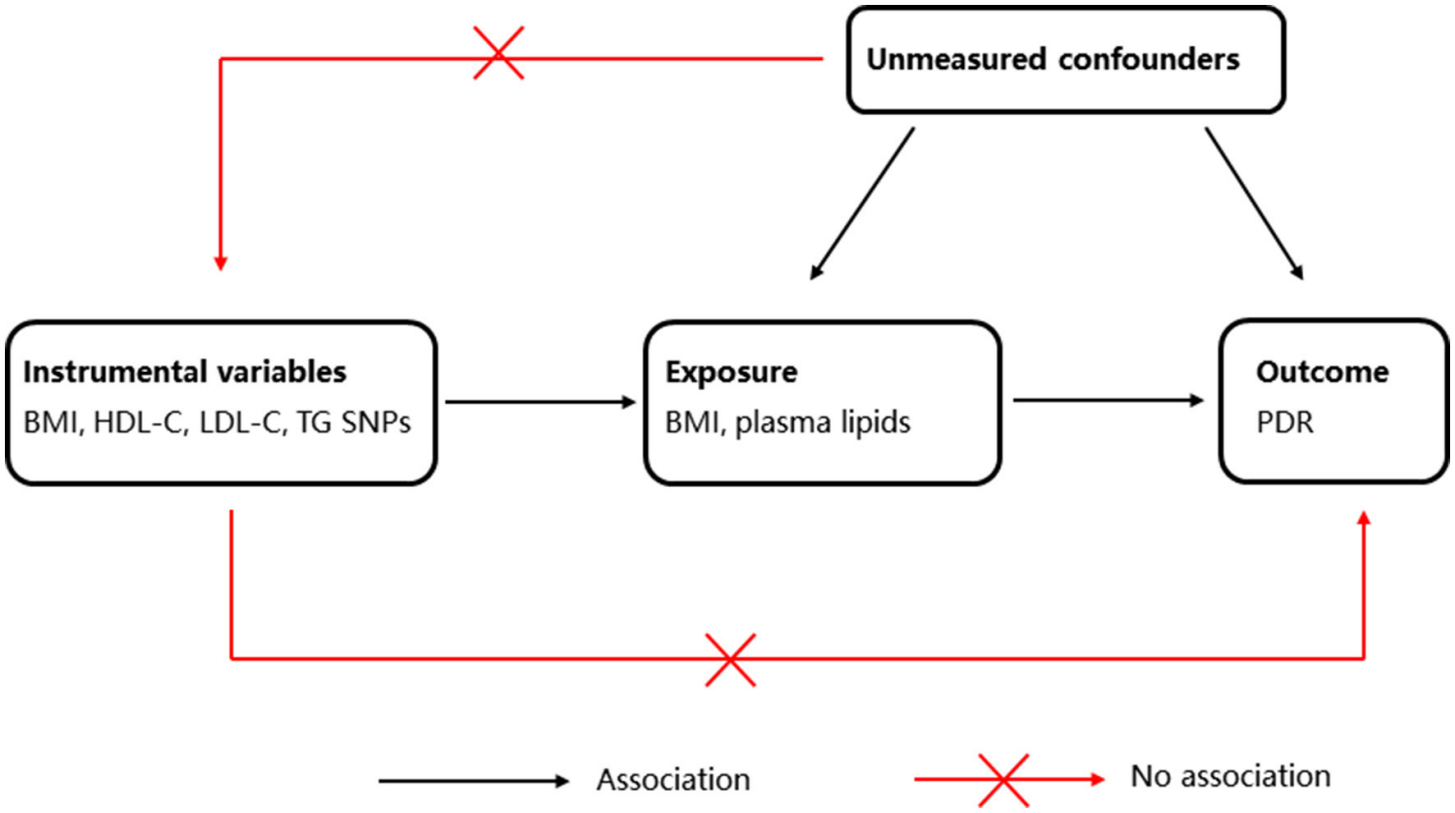
Schematic representation of Mendelian randomization (MR) analysis. BMI, body mass index; HDL-C, high-density lipoprotein cholesterol; LDL-C, low-density lipoprotein cholesterol; TG, triglyceride; PDR, proliferative diabetic retinopathy.

To investigate the causal relationship between BMI, plasma lipid levels, and risk of PDR, we applied the two-sample MR, an approach to determine causal effects using GWAS summary statistics for exposure and outcome from separate GWASs. The MR study design is illustrated in Figure 1.

The data and information we used in this article were all searched and downloaded from the public database. No ethical review was required for this study.

### Genetic instruments

Two-sample MR analyses were performed using GWAS summary data. BMI and three plasma lipids—high-density lipoprotein cholesterol (HDL-C), low-density lipoprotein cholesterol (LDL-C), and triglyceride (TG)—were included in this study as the exposure variables. Publicly accessible data for genetic variants associated with BMI and three plasma lipids were obtained from GWAS summary datasets generated by many different consortia and were deposited in the MR-Base database (https://gwas.mrcieu.ac.uk/). The FinnGen Biobank for PDR is available at https://r5.finngen.fi/.

The datasets included 99,998 European participants and the number of included SNPs was 7,191,606 for BMI, 77,409 participants and 7,892,377 SNPs for HDL-C, 70,814 participants and 7,892,997 SNPs for LDL-C, and 78,700 participants and 7,892,037 SNPs for TG. The GWAS summary data for PDR from the FinnGen biobank included 2,12,889 participants of European ancestry (8,681 cases and 204,208 controls). The number of SNPs included in the study was 16,380,460 (Supplementary Table 1).

All SNPs with *p*-values ≤ 5.0 × 10^−8^ were extracted, and then we used linkage disequilibrium (LD) to minimize the impact. We set the threshold of statistical significance as “distance> 10,000 kb, r^2^ < 0.001” to identify the SNPs associated with exposure. These SNPs were used as instrumental variables (IVs) in the MR analysis.

### Mendelian randomization

We performed UVMR analyses for BMI and plasma lipid levels on PDR risk separately to estimate the total causal effects of BMI on PDR and plasma lipid levels on PDR. To assess the causal effect of BMI on PDR, the inverse variance-weighted (IVW) method was used as the main MR analysis, and other methods, such as MR–Egger, weighted median, simple mode, and weighted mode, were also used as complementary analyses. A *P*-value of <0.05 was considered to be statistically significant.

To explore whether PDR had any causal effect on the BMI, we also performed a reverse MR analysis using the SNPs associated with PDR as IVs (i.e., PDR as exposure and the identified causal BMI as outcome).

To investigate the direct effects of each, we applied MVMR to identify whether the effect of BMI and HDL-C is independent of each other on PDR risks. The SNPs used to conduct MVMR were combinations of IVs of each exposure (duplicates were excluded). The SNP for each exposure has been described in the methods of genetic instruments. We used the IVW method to estimate causal effects in MVMR analysis.

According to Bonferroni correction analysis, a *P*-value of <0.025 was considered to be statistically significant in MVMR analyses.

### Sensitivity analysis

Instrument strength for each candidate SNP in MR was estimated using the F-statistic. We evaluated the SNP power using the F statistics (12) (F = Beta^2^/SE^2^) for each SNP, and F > 10 was viewed as a strong instrument.

Heterogeneity was tested by Cochran's Q test and I^2^ statistics (13) (I^2^ = 100%^*^(Q-df)/Q, where Q represents the quantitative value of Cochran's Q test and df represents the degree of freedom which equals to the number of instrumental variables used minus one) to evaluate the stability and reliability of MR results. A *p*-value of < 0.05 or an I^2^ value of >50% was regarded as statistically significant (14). The intercept of the Egger model can be used for the statistical test of pleiotropy (15), where deviation from 0 denotes the presence of directional pleiotropy. In addition, the MR-PRESSO test can detect possible outliers, and the MR-PRESSO global test can be used to identify the potential horizontal pleiotropic effects of the SNPs (16). A leave-one-out sensitivity test was utilized to examine the effect of individual SNPs on the causal inference. We removed the single SNPs one by one and calculated the effect of the remaining SNPs by the IVW method (17).

UVMR and MVMR analyses were performed in R (version 4.2.1) using the “TwoSampleMR” version 0.5.6 and “MR-PRESSO” package (18) (https://mrcieu.github.io/TwoSampleMR/). A *P*-value <0.05 was considered to indicate nominal significance.

## Results

### MR analysis of BMI and risk of PDR

In total, 35 SNPs for BMI (Supplementary Table 2) were selected as the genetic instruments. We calculated F-statistics to evaluate the strength of the instrumental variable effect. The rule-of-thumb of F was set as 10 to avoid bias.

**Figure 2:**
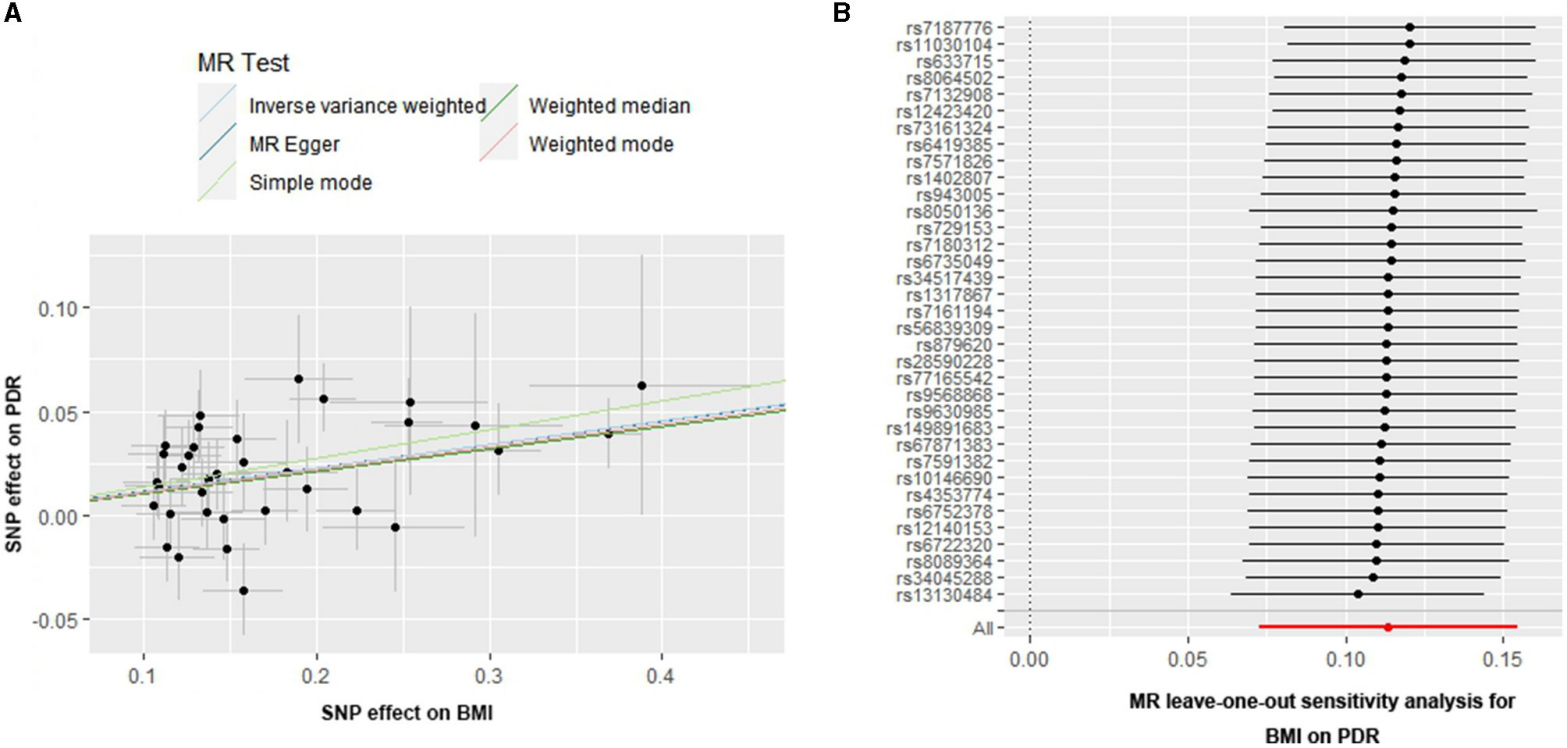
Scatter and leave-one-out plots for BMI on the risk of PDR. **(A)** Scatter plots of the genetic associations of BMI-associated SNPs against the genetic associations of PDR. The slopes of each line represent the causal association for each method. **(B)** Leave-one-out analysis plots for BMI on PDR risk.

The causal associations of genetically predicted BMI with PDR presented a positive result, which means that higher BMI was associated with a higher risk of PDR [odds ratio (OR) = 1.120, 95% confidence interval (CI) = 1.076–1.167, *P* < 0.001, using the IVW method] (Table 1) (Figure 2A). The sensitivity analysis showed that there were no heterogeneities (IVW: Q-value = 39.657; df = 34; *P* = 0.232; I^2^ = 14.26%; MR Egger: Q-value = 39.656; df = 33; *P* = 0.198; I^2^ = 16.78%) and no directional pleiotropies (MR–Egger intercept = 2.942 × 10^−4^; SE = 0.010; *P* = 0.976).

**Table 1:** Five MR results of BMI and plasma lipids on risk of PDR.

**Variable exposure**	**nSNP**	**OR**	**95%CI**	***P*-value**
**BMI**				
MR egger	35	1.118	0.999–1.252	0.061
Weighted median	35	1.113	1.050–1.180	< 0.001
IVW	35	1.120	1.076–1.167	< 0.001
Simple mode	35	1.147	1.025–1.284	0.023
Weighted mode	35	1.115	1.033–1.204	0.009
**HDL-C**				
MR egger	65	0.978	0.815–1.172	0.806
Weighted median	65	0.966	0.838–1.114	0.637
IVW	65	0.898	0.811–0.995	0.040
Simple mode	65	0.902	0.690–1.181	0.457
Weighted mode	65	0.947	0.822–1.090	0.447
**LDL-C**				
MR egger	43	0.908	0.785–1.051	0.202
Weighted median	43	0.949	0.841–1.072	0.402
IVW	43	0.933	0.848–1.027	0.156
Simple mode	43	0.964	0.786–1.180	0.722
Weighted mode	43	0.964	0.858–1.082	0.532
**TG**				
MR egger	37	1.034	0.678–1.579	0.876
Weighted median	37	0.922	0.761–1.118	0.409
IVW	37	1.009	0.814–1.251	0.932
Simple mode	37	0.993	0.653–1.506	0.972
Weighted mode	37	0.839	0.626–1.124	0.246

PDR, proliferative diabetic retinopathy; HDL-C, high-density lipoprotein cholesterol; LDL-C, low-density lipoprotein cholesterol; TG, triglyceride; IVW, inverse variance weighted; MR, Mendelian randomization; OR, odds ratio; nSNP, number of single nucleotide polymorphism.

In addition, the MR-PRESSO global test (P for global test = 0.355) supports this result. Moreover, the leave-one-out test suggested that the observed associations may not be driven by any single SNP (Figure 2B), indicating that the results were reliable and stable.

In reverse MR analysis, there was no evidence of a causal effect of PDR on BMI (OR = 0.91, 95%CI = 0.79–1.05, *P* = 0.18, using the IVW method).

### MR analysis of plasma lipid levels and risk of PDR

We further investigated the relationship between plasma lipid levels and the risk of PDR (Table 1). In total, 65 SNPs for HDL-C (Supplementary Table 3), 43 SNPs for LDL-C, and 37 SNPs for TGs were selected as the genetic instruments.

**Figure 3:**
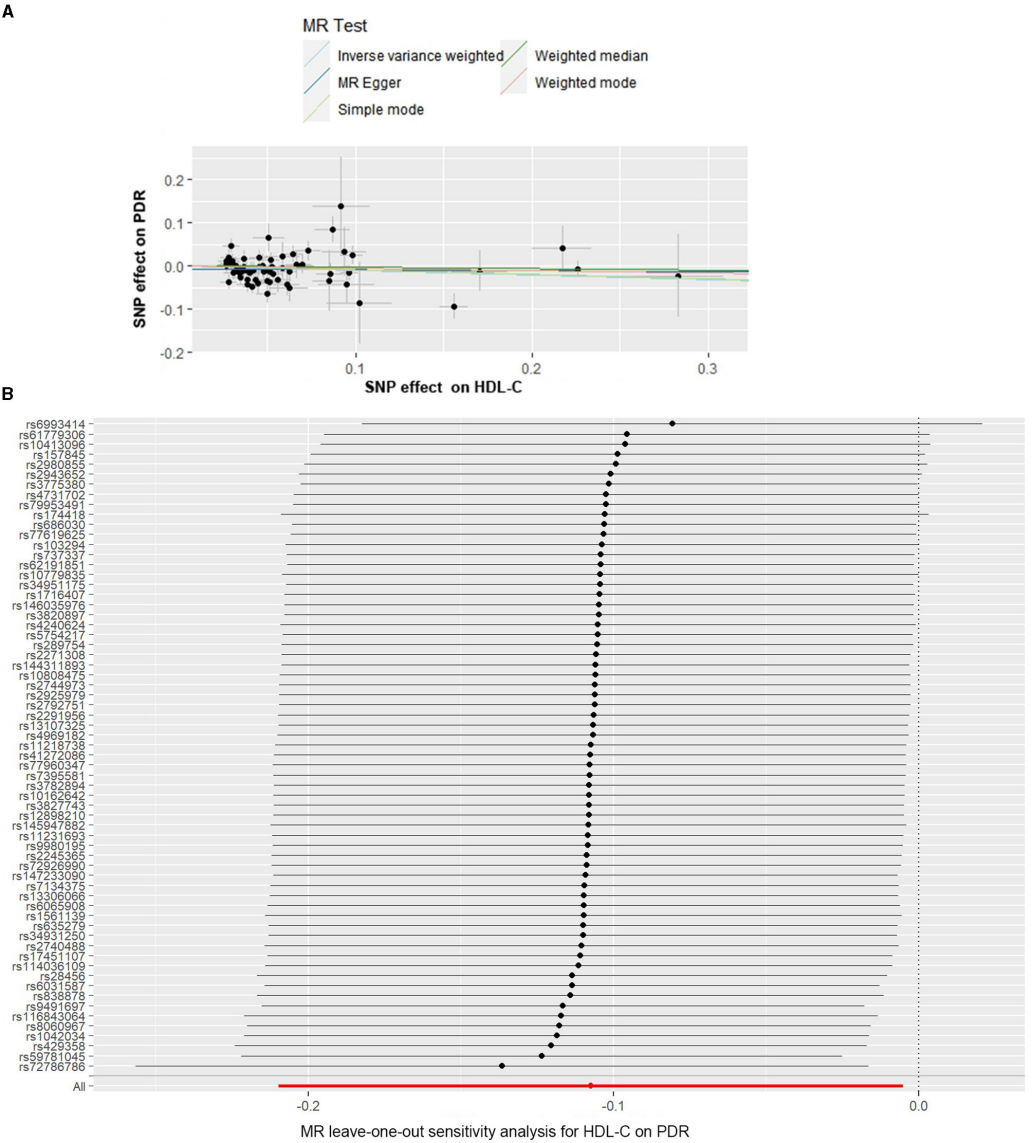
Scatter and leave-one-out plots for HDL-C on the risk of PDR. **(A)** Scatter plots of the genetic associations of HDL-C-associated SNPs against the genetic associations of PDR. The slopes of each line represent the causal association for each method. **(B)** Leave-one-out analysis plots for HDL-C on PDR risk.

The causal associations of genetically predicted HDL-C levels with PDR based on the IVW method showed that the lower HDL-C levels were associated with a higher risk of PDR (OR = 0.898, 95% CI = 0.811–0.995, *P* = 0.040) (Table 1, Figure 3A). The MR-Egger intercept test suggested a low probability of directional pleiotropy for HDL-C (intercept = −6.249 × 10^−3^; SE = 5.691 × 10^−3^; *P* = 0.276). Although the results of Cochran's Q (Q-value = 100.583; df = 63; *P* = 0.0024, by IVW) showed there was heterogeneity, I^2^ = 37.37% suggested that it did not have significant heterogeneity. We still used the random effects IVW MR analysis (*P* = 0.040), Bowden et al. (19) indicating that a higher HDL-C level was associated with a lower risk of PDR. The result of the MR analysis was driven by potentially influential SNPs identified in the “leave one out” analysis, and we should carefully interpret the result and draw a cautious conclusion (Figure 3B).

When we assessed the causal relationship between LDL-C and PDR, no evidence of an association was detected using the IVW analysis method (*P* = 0.156) (Table 1; Supplementary Figure 1). The MR-Egger intercept test did not reveal signs of horizontal pleiotropy for LDL-C (intercept = 2.884 × 10^−3^, SE = 5.910 × 10^−3^, *P* = 0.628). Non-significant heterogeneity was detected across the instrument SNP effects (Q = 57.027; *P* = 0.061, by IVW). Furthermore, in the leave-one-out sensitivity analysis, we found that no single SNP significantly influenced the overall effect of LDL-C on PDR (Supplementary Figure 2).

In the UVMR analysis of TG and PDR, the causal effect estimated from five methods all suggested a negative result (*P* > 0.05) (Table 1; Supplementary Figure 3). No statistical evidence of horizontal pleiotropy was found across TGs (intercept = −1.673 × 10^−3^, SE = 0.013, *P* = 0.895). Furthermore, no significant distortion was observed in the causal estimates before and after outlier removal (MR-PRESSO distortion test, *p* = 0.855). Although heterogeneity in the SNP effects was indicated by the Cochran's *Q* test (Q = 134.209, *P* = 3.053 × 10^−13^), plots of leave-one-out analysis that are shown in Supplementary Figure 4 demonstrated no consistent causal relationship between genetically predicted TG and PDR. Thus, this suggests that there is stability in our results.

### MVMR analysis

Moreover, we carried out IVW MVMR analyses to estimate the direct causal effect of genetically predicted BMI and HDL-C levels on PDR risk. In the MVMR analysis controlling for BMI, there was no evidence for a direct causal effect of the HDL-C level on the risk of PDR (IVW OR = 0.911, 95% CI = 0.823, 1.008, *P* = 0.072). After adjusting for the HDL-C level, there was strong evidence for a direct causal effect of BMI on the risk of PDR (IVW OR = 1.106, 95% CI = 1.049, 1.166, *P* < 0.001) (Supplementary Table 4).

## Discussion

In the present study, we utilized both UVMR and MVMR analyses to estimate a causal and independent effect of BMI and plasma lipid level on PDR risk. Our study provided robust evidence that BMI was responsible for deleterious effects on higher PDR risk, and BMI may account for the protective effects of HDL-C against the risk of PDR, whereas no clear evidence for an independent causal effect of HDL-C on the risk of PDR. By using MVMR analysis, we found that BMI can still directly influence PDR after adjustment for HDL-C. The results of reverse MR analysis showed no evidence of reverse causality from PDR to BMI. Moreover, several sensitivity analyses of our results are essentially robust, including heterogeneity tests, pleiotropy tests, and leave-one-out sensitivity tests.

The appearance of retinal neovascularization means a critical change in the progression of DR, the sight-threatening endpoint (PDR). Visual loss can occur suddenly because of vitreous hemorrhage (VH) or tractional retinal detachment (TRD) due to progressive fibrosis (1). In this stage, it is necessary to intervene with some treatments, such as laser photocoagulation and intraocular injection of anti-VEGF agents, and even vitreoretinal surgery may occasionally be needed. Therefore, it is crucial to explore the modifiable risk factors for PDR, which is why we chose PDR rather than DR as the outcome of our study.

It is widely acknowledged that obesity correlates with an increased risk for hypertension, stroke, coronary atherosclerotic heart disease, and diabetes. Moreover, the influence of obesity on eye diseases has been widely confirmed, such as primary open-angle glaucoma (20), age-related maculopathy (21), and cataracts (22). In the study, obesity was judged by BMI, and three kinds of plasma lipid levels were included for analysis simultaneously. To date, various studies have focused on the mechanism between BMI and DR. Miyazawa- Miyazawa-Hoshimoto et al. (23) found that VEGF concentrations were positively correlated with BMI, while VEGF has been shown to be involved in the pathogenesis of PDR. This is consistent with our results that a higher BMI was associated with a greater risk of PDR. Considine et al. (24) found that there was a strong positive correlation between serum leptin concentrations and BMI. Some studies have suggested that serum leptin plays a role in the pathogenesis of DR. Uckaya et al. (25) found that the plasma level of leptin was significantly higher in PDR than that in patients with non-PDR or without retinopathy. In addition, the result that vitreous leptin concentrations were especially higher in patients with PDR supports the conclusions above (26). This phenomenon may be involved in the mechanism of leptin-induced promotion of angiogenesis and neovascularization (27). In addition, BMI is often correlated with hypertension and dyslipidemia, which are correlated with DR. All the results have supported a relationship between BMI and PDR. Moreover, central obesity was defined by waist circumference (WC) or waist-to-hip ratio (WHR). Zhou et al. (28) found that WC is associated with the risk of DR in the Chinese population. Man et al. (29) found that a higher WHR was associated with the presence and severity of DR in women. Ranganathan et al. (30) also supported this finding, and they found that increased waist circumference was a risk factor for diabetic retinopathy in type 2 diabetes patients older than 45 years.

The role of dyslipidemia in DR has now attracted increasing attention although the results remain controversial. Previous studies have shown an association between dyslipidemia and DR, but the causal effect remains unclear. Yau et al. (31) found that higher total serum cholesterol was associated with a higher risk of DME and confirmed the importance and influence of dyslipidemia, hyperglycemia, and hypertension as major modifiable risk factors for the risk of all DR, whereas Wong et al. (32) published the conflicting reports about this risk factor, showing that higher total cholesterol levels were associated with reduced odds of DR. The study by Zhang et al. (33) provided strong evidence that dyslipidemia promotes the development of DR by increased secretion of VEGF-A, VEGF-C, VEGF-D, and PlGF in patients with DR. On the other hand, elevated lipids are associated with endothelial dysfunction, causing hemodynamic changes, retinal tissue hypoxia, and microcirculatory disorders, and with breakdown of the blood-retinal barrier, which causes the development of DR (4). Although our results did not show a clear causal relationship between plasma lipids and PDR, the mechanism behind them still deserves further investigation.

MR was first proposed and used in 1991 (34), and then it became a greatly powerful approach to identify the causal effect of exposure on the outcome in epidemiology. The core of MR is the use of IVs, which must be associated with the exposure and only affect the outcome via the exposure, while there is no factor that can cause both the IVs and the outcome (35). Our study had some strengths as follows: First, we used GWAS summary data to extract SNPs for exposure and selected the latest studies and the largest SNP database, which means that the results were reliable. Second, the law of independent assortment means that the genetic variations related to exposure are randomly distributed among the population at birth and, thus, compared with traditional observational research and RCTs, the causal association of BMI, plasma lipids, and PDR was not distorted by confounding factors. Third, our results were robust according to the results of sensitivity analysis. However, there are several limitations in our study. First, only participants of European ancestry were included in our study; thus, it is unclear whether our results are also applicable to other populations. Further investigation is needed in other ethnic groups. Second, although several sensitivity analyses of our results are essentially robust, there may be unidentified pleiotropic effects in these datasets, so future investigators should analyze more MR methods and collect more data to minimize and avoid bias. Finally, although our results indicated that BMI is associated with an increased risk of PDR, the mechanisms are not entirely clear.

In summary, our results suggested a significant influence of BMI on PDR risks, providing genetic evidence that higher BMI is related to a higher risk of PDR. No clear evidence was found for an independent causal effect of HDL-C on PDR risk. Moreover, further studies are needed to investigate the underlying mechanism of BMI for prevention and therapeutic treatment in PDR. Our results provide a new research field for the effective screening and management of PDR risk factors.

## Data availability statement

The original contributions presented in the study are included in the article/Supplementary material, further inquiries can be directed to the corresponding authors.

## Author contributions

YShu, QZ, and YShao designed the study and drafted the manuscript. HL and SQ analyzed the data. WH and XL reviewed and edited the manuscript. YB led and funded the project and overall supervised the project. YB and QZ discussed, revised, and finalized the manuscript. All authors approved the final version of the manuscript to be published.
