# Supplementary material for: BMI and plasma lipid levels with risk of proliferative diabetic retinopathy: a univariable and multivariable Mendelian randomization study
Source: Front Nutr. 2023 Sep 13;10:1099807. doi: 10.3389/fnut.2023.1099807 (PMC10524610; doi:10.3389/fnut.2023.1099807)
Supplement: Supplementary file 1 [file Data_Sheet_1.doc]

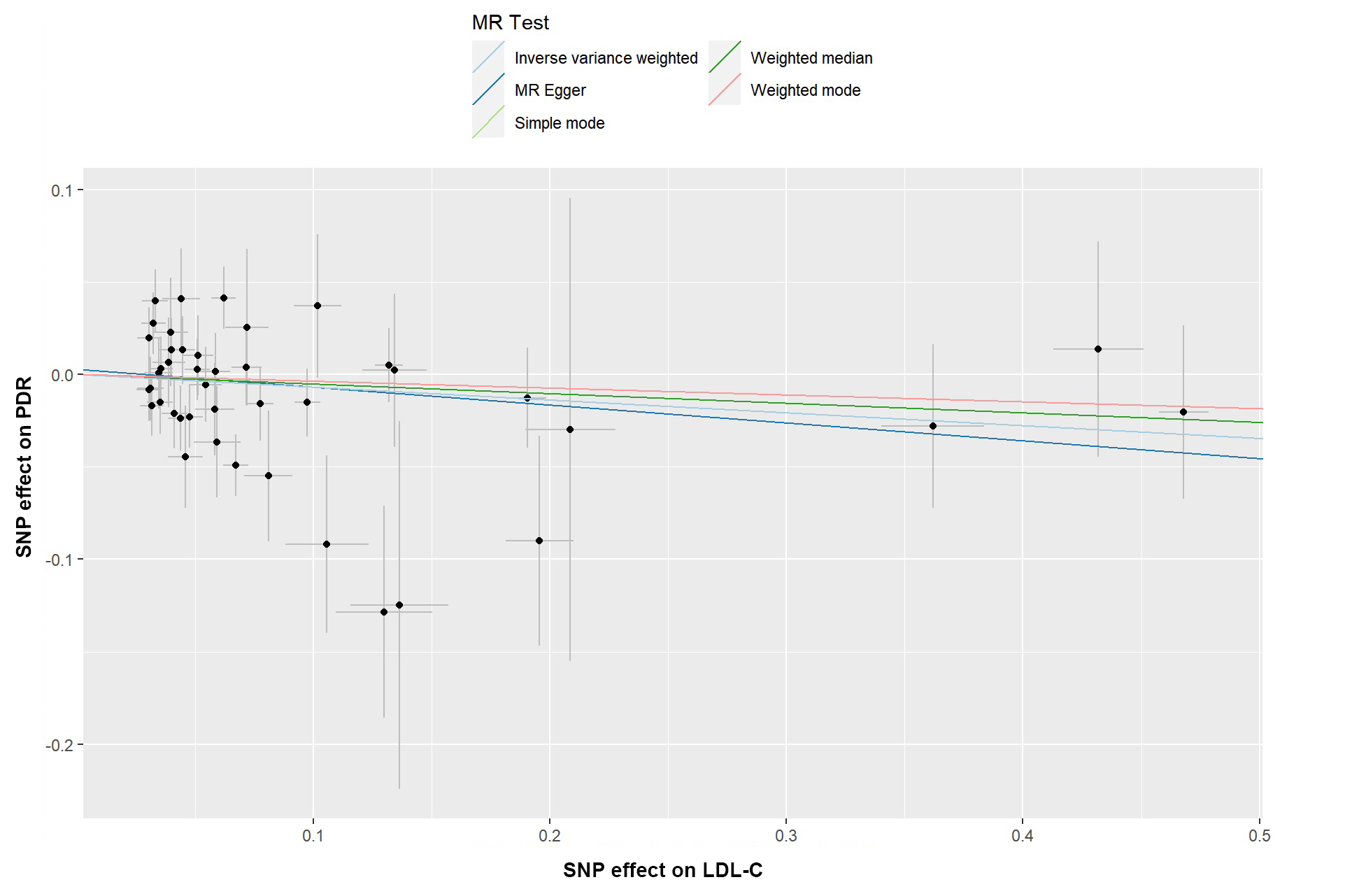


Figure 1. Scatter plots of the genetic associations of LDL-C associated SNPs against the genetic associations of PDR. The slopes of each line represent the causal association for each method.


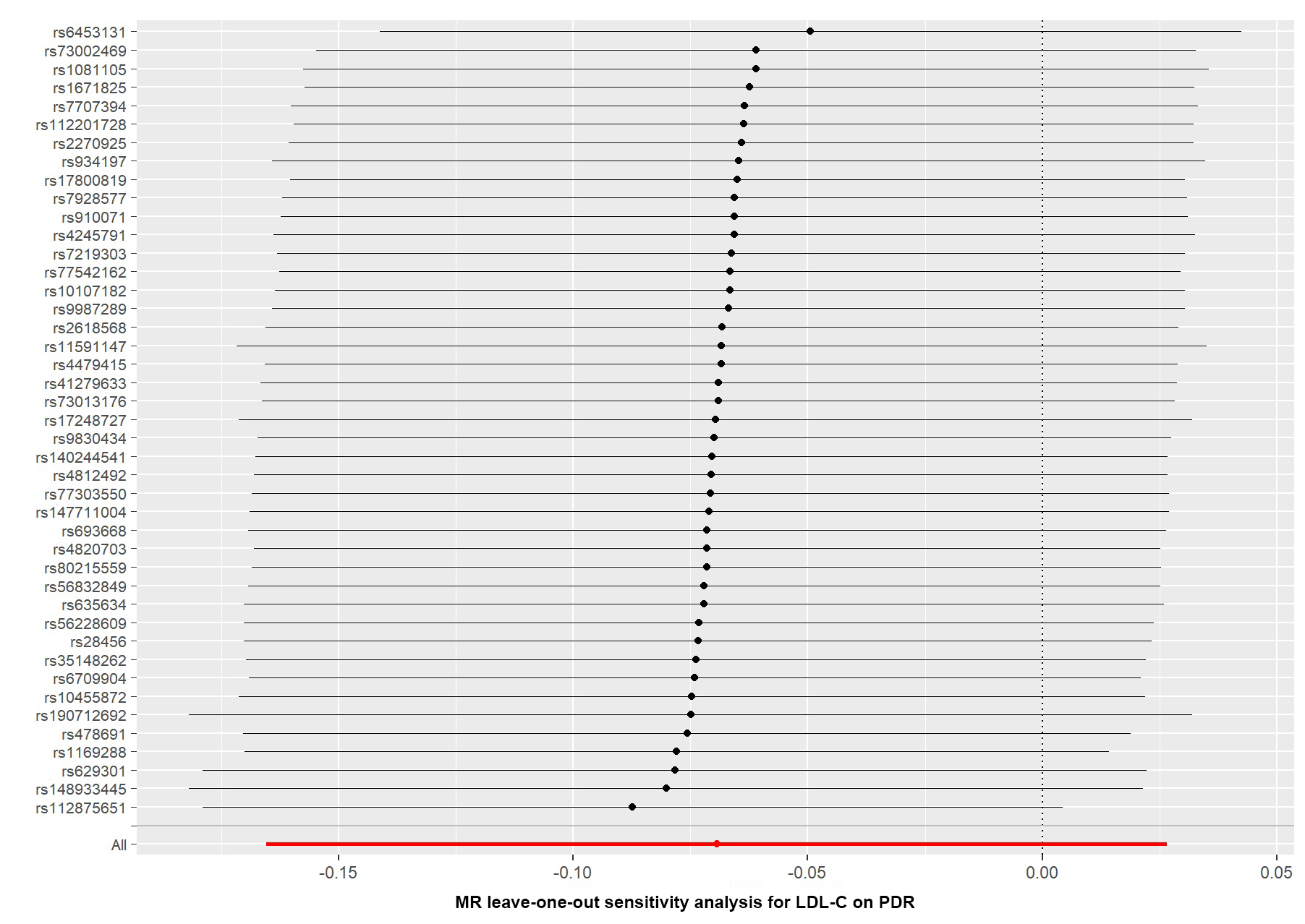


Figure 2. Leave-one-out analysis plots for LDL-C on PDR risk.


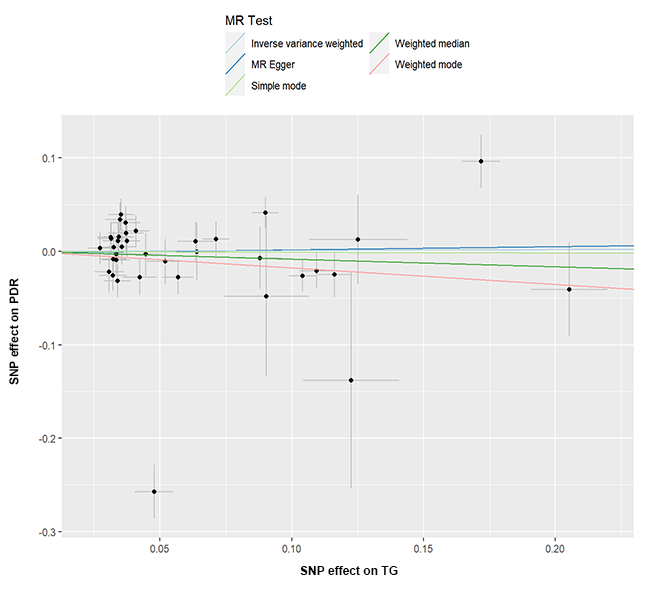


Figure 3. Scatter plots of the genetic associations of TG associated SNPs against the genetic associations of PDR. The slopes of each line represent the causal association for each method.


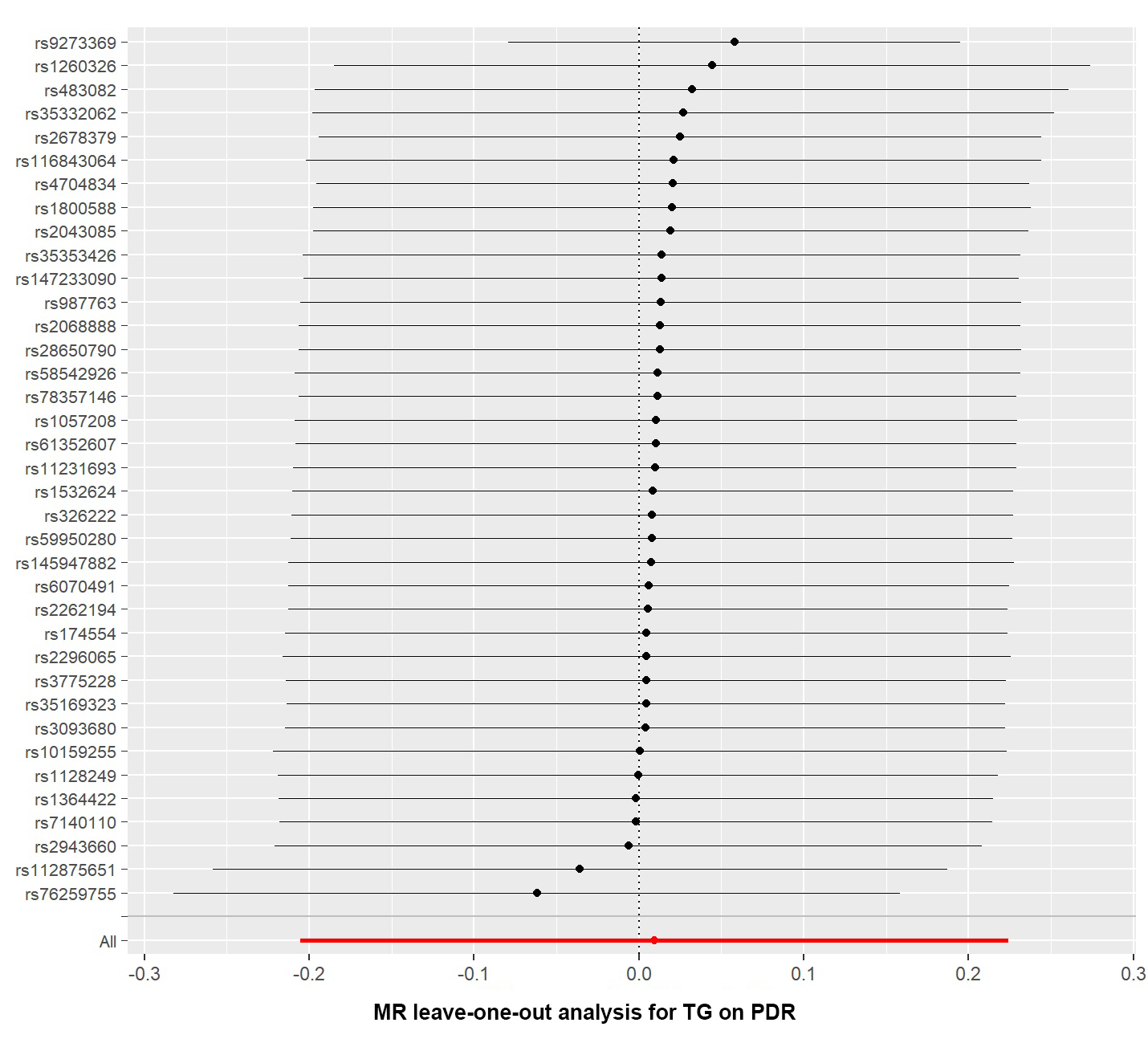


Figure 4. Leave-one-out analysis plots for TG on PDR risk.
